# Simple algorithm for judging equivalence of differential-algebraic equation systems

**DOI:** 10.1038/s41598-023-38254-y

**Published:** 2023-07-17

**Authors:** Shota Kato, Chunpu Zhang, Manabu Kano

**Affiliations:** Department of Systems Science, Kyoto University, Yoshida-honmachi, Sakyo-ku, Kyoto, 606-8501 Japan

**Keywords:** Chemical engineering, Computer science, Information technology

## Abstract

Mathematical formulas play a prominent role in science, technology, engineering, and mathematics (STEM) documents; understanding STEM documents usually requires knowing the difference between equation groups containing multiple equations. When two equation groups can be transformed into the same form, we call the equation groups equivalent. Existing tools cannot judge the equivalence of two equation groups; thus, we develop an algorithm to judge such an equivalence using a computer algebra system. The proposed algorithm first eliminates variables appearing only in either equation group. It then checks the equivalence of the equations one by one: the equations with identical algebraic solutions for the same variable are judged equivalent. If each equation in one equation group is equivalent to an equation in the other, the equation groups are judged equivalent; otherwise, non-equivalent. We generated 50 pairs of equation groups for evaluation. The proposed method accurately judged the equivalence of all pairs. This method is expected to facilitate comprehension of a large amount of mathematical information in STEM documents. Furthermore, this is a necessary step for machines to understand equations, including process models.

## Introduction

The volume of scientific literature has been increasing exponentially, and this trend continues with an average doubling period of 15 years^1^ and is expected to continue. When writing a report on a particular topic, such as a review of previous studies, it is necessary to survey the increasing amount of literature. The key to understanding multiple documents and organizing the information is to recognize the difference among the documents, which requires much toil. Automatically judging the equivalence of the information would be helpful for efficiently processing a large number of documents.

Equations representing the relationships between variables play a central role in understanding documents of science, technology, engineering, and mathematics (STEM). Multiple equations are often used as a single entity to describe the relationship between variables. It is, therefore, crucial to recognize the difference between equation groups consisting of two or more equations when understanding STEM documents. A physical model is a typical example of an equation group. For example, suppose a researcher wants to build a physical model. Before building the model, the researcher surveys previous studies and identifies the differences among the multiple models in the previous studies, which is an arduous task. Several studies have dealt with a text in the chemical engineering field using natural language processing techniques^2–4^, but no studies have aimed to reduce this kind of effort.

Since a variable is sometimes expressed by different symbols among documents, we have to extract variable definitions^5–7^ and unify the variable symbols’ representations^8^ before judging the equivalence of equation groups. Such a method can be developed independently of the equivalence judgment; thus, we assume that different symbols do not represent the same variable in this study.

In order to judge the equivalence of equation groups, computers must grasp the meanings of mathematical formulas. Converting natural language into vectors is one of the methods for computers to handle the meanings of natural language, and recent studies utilize neural network models, such as Word2Vec^9^, Transformer^10^, and Bidirectional Encoder Representations from Transformers (BERT)^11^. Similarly, several studies represent mathematical formulas with neural network models^12–14^. Mansouri et al.^12^ defined a similarity between two formulas based on their appearances, but similar-looking formulas do not necessarily perform the same calculation. For example, the similarity between $a+b=0$ and $a-b=0$ is higher than that between $a+b=0$ and $a=-b$ based on the models by Mansouri et al.^12^. The existing neural network models, which focus on the appearance of formulas, do not work for the equivalence judgment of equation groups.

Another approach for handling the meanings of mathematical formulas in computers is encoding the formulas with special markup languages such as Content Mathematical Markup Language (MathML)^15^ and OMDoc^16^. However, such markup is rarely used to publish mathematical knowledge^17^. The commonly used methods for notating mathematical expressions are LaTeX for papers and Presentation MathML^15^ for the Web.

The most effective way for computers to comprehend formulas’ meanings is to use computer algebra systems (CASs). Formula transformations, for example, from LaTeX to the format usable in CASs, are well-studied in the CASs literature^18,19^. Further, CASs, such as Mathematica and Maple, have LaTeX input support.

CASs can solve equations and judge the equivalence of two equations by comparing their solutions for one variable. Similarly, if two equation groups are solvable for one variable, CASs can judge their equivalence by comparing their solutions. However, when one of two equation groups to be compared is not solvable for one variable, CASs alone cannot correctly judge their equivalence. Physical models are commonly represented by combinations of differential equations and algebraic equations, called differential-algebraic equation (DAE) systems. Therefore, it is essential to address this issue for machines to compare equation groups contained in documents related to chemical engineering.

In this study, we propose a method for solving this problem. The proposed method uses a computer algebra system to eliminate variables contained only in either equation group and judge whether each equation in one equation group is equivalent to an equation in the other. We generate 50 equivalent and non-equivalent pairs of equation groups and evaluate the performance of our proposed method.

## Methods

### Equivalence judgment methods

**Figure 1:**
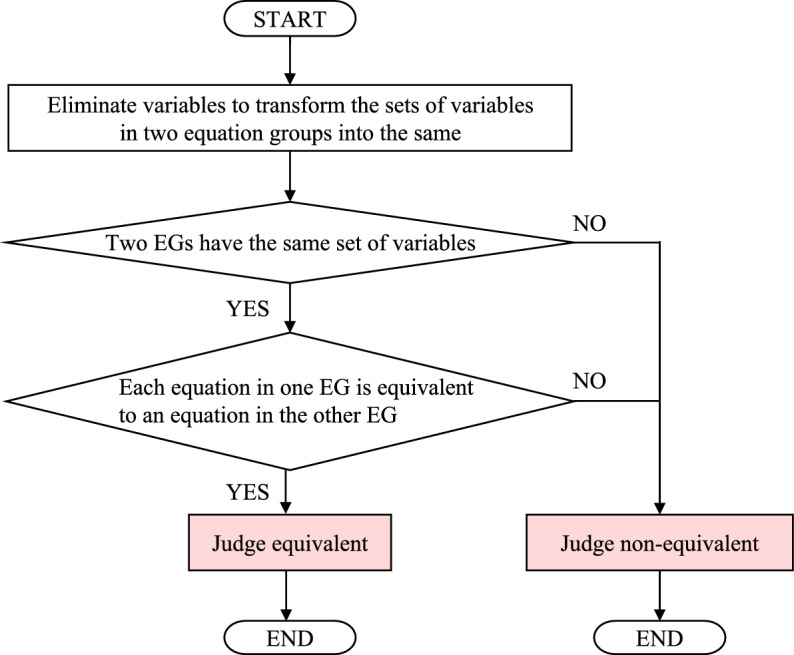
A schematic illustration of the algorithm for judging the equivalence of two equation groups. EG represents an equation group.

Figure 1 schematically presents our proposed method for judging the equivalence of two equation groups. The algorithm 1) eliminates variables, 2) checks whether two equation groups have the same set of variables, and 3) judges the equivalence between each equation in one equation group and each equation in the other equation group. If two equation groups share the same set of variables after variable elimination and each equation in one equation group is equivalent to an equation in the other, the equation groups are judged equivalent; otherwise, they are judged non-equivalent.

Our algorithm utilizes a CAS to (1) solve equations, (2) substitute formulas into variables to eliminate the variables, and (3) judge whether two formulas are equivalent.

In this study, we assume that two equivalent equations are solvable for any variable, and each number of solutions for the variable is one, that is, the solution for the variable is unique.

#### Equivalence judgment of equations

Two equivalent equations have to satisfy the following requirements:

- The equations have the same set of variables.
- The solutions of the equations for any variable are the same.

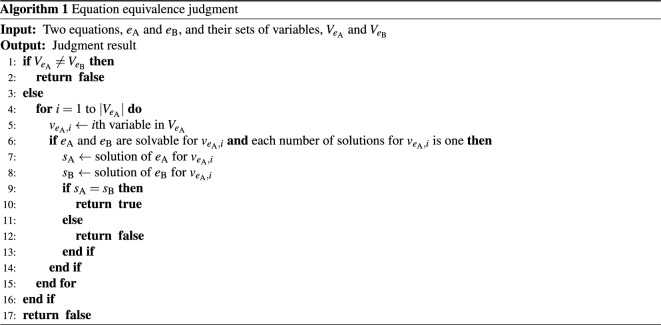

We propose Algorithm 1 to sequentially check the above points and judge the equivalence of two equations, $e_{\textrm{A}}$ and $e_{\textrm{B}}$. In Algorithm 1, the sets of variables of $e_{\textrm{A}}$ and $e_{\textrm{B}}$, $V_{e_{\textrm{A}}}$ and $V_{e_{\textrm{B}}}$, are compared at first (Lines 1 and 2). The algorithm then checks whether $e_{\textrm{A}}$ and $e_{\textrm{B}}$ are solvable for any variable, and each number of solutions for the variable is one (Line 6). Here, ‘solvable’ means that a variable can be explicitly expressed by the other variables. If the solutions of $e_{\textrm{A}}$ and $e_{\textrm{B}}$, $s_{\textrm{A}}$ and $s_{\textrm{B}}$, are the same, the two equations are judged equivalent (Lines 7–10). The judgment was conducted by converting $s_{\textrm{A}}$ and $s_{\textrm{B}}$ to simple forms by conversion rules implemented in CAS, such as Sympy’s simplify^20^ and Mathematica’s Simplify^21^, and determining if the obtained two formulas are the same. The equations are judged non-equivalent if any variable’s solutions are different (Lines 11 and 12), either equation cannot be solved, or the number of solutions is more than one (Line 17).

#### Variable elimination in equation group

Before judging the equivalence of two equation groups, we eliminate variables in each equation group so that the equation groups have the same set of variables. For example, equation groups $\{ x + y = 0\}$ and $\{ x=t, y=-t\}$ are easily judged to be equivalent when the variable *t* is removed from the latter equation group. A variable is eliminated by solving an equation for the variables and substituting its solution to the other equations with the variable as shown in Algorithm 2.
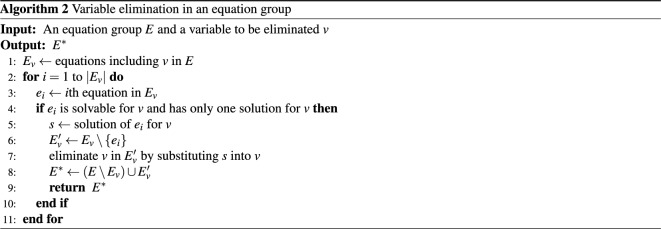


To eliminate a variable *v* in an equation group *E*, the algorithm first obtains the equations including *v* in *E*, $E_{v}$ (Line 1). If the *i*th equation in $E_{v}$, $e_{i}$, is solvable for *v* and has only one solution for *v*, the solution of $e_i$ for *v*, *s*, is computed (Lines 3–5). Then, *s* is substituted into *v* in all equations in $E_v$ except $e_i$, and the set of the substituted equations $E_v'$ is obtained (Lines 6 and 7). Finally, the set of equations $E^*$ that does not include *v* is derived by replacing $E_v$ in *E* with $E_v'$ (Line 8).

#### Equivalence judgment of equation groups

We judge not only the equivalence between two equations but also the equivalence between two equation groups consisting of multiple equations. Equivalent two equation groups after variable elimination need to satisfy the following two conditions:

- The two equation groups share the same set of variables.
- Each equation in one equation group is equivalent to an equation in the other equation group.

Based on these conditions, we propose Algorithm 3 for equivalence judgment of two equation groups $E_{\textrm{A}}$ and $E_{\textrm{B}}$, whose sets of variables are $V_{E_{\textrm{A}}}$ and $V_{E_{\textrm{B}}}$, respectively.
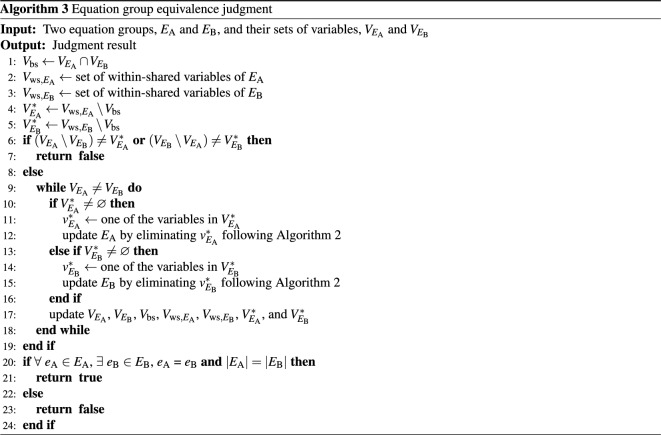


At first, the algorithm compares $V_{E_{\textrm{A}}}$ and $V_{E_{\textrm{B}}}$ to transform them into the same (Lines 1–19). Here, we define between-shared variables $V_{\textrm{bs}}$ as the variables shared between $E_{\textrm{A}}$ and $E_{\textrm{B}}$, and within-shared variables $V_{\textrm{ws}, E_i}$ as the variables shared between the equations within an equation group $E_i$ (Lines 1–3). $V_{E_{\textrm{A}}}$ and $V_{E_{\textrm{B}}}$ are transformed into the same by eliminating the variables appearing only in either equation group. Besides, variables that can be eliminated in an equation group are included in its within-shared variables. Hence, the variables to be eliminated in $E_{\textrm{A}}$ and $E_{\textrm{B}}$, which are denoted by $V_{E_{\textrm{A}}}^*$ and $V_{E_{\textrm{B}}}^*$, are the set difference of $V_{\textrm{ws}, E_{\textrm{A}}}$ and $V_{\textrm{bs}}$ and that of $V_{\textrm{ws}, E_{\textrm{B}}}$ and $V_{\textrm{bs}}$, respectively (Lines 4 and 5). When the variables appearing only in either equation group are not equal to the variables to be eliminated, $V_{E_{\textrm{A}}}$ and $V_{E_{\textrm{B}}}$ cannot be transformed into the same. In such a case, $E_{\textrm{A}}$ and $E_{\textrm{B}}$ are judged non-equivalent (Lines 6 and 7). Otherwise, the variables in $V_{E_{\textrm{A}}}^*$ and $V_{E_{\textrm{B}}}^*$ are eliminated one by one until the two equation groups share the same set of variables (Lines 8–18).

After the variable elimination, the algorithm checks whether each equation in $E_{\textrm{A}}$ is equivalent to an equation in $E_{\textrm{B}}$ and each number of the equations is the same. If all the equations in $E_{\textrm{A}}$ and $E_{\textrm{B}}$ have a one-to-one relationship, the two equation groups are judged to be equivalent; otherwise, non-equivalent (Lines 20–24).

**Figure 2:**
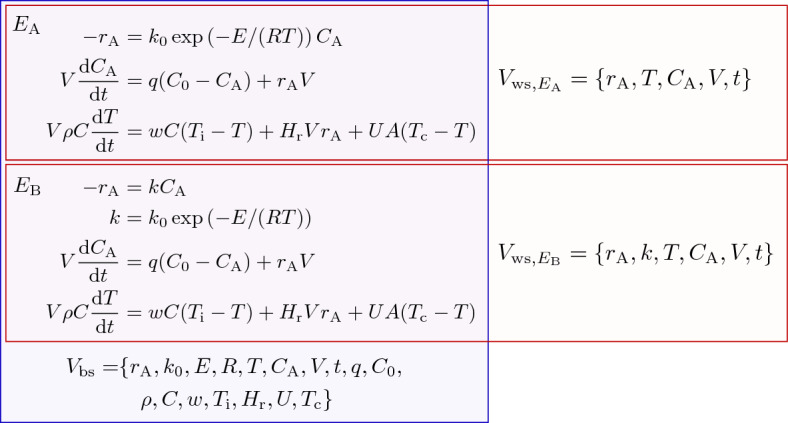
An example two equivalent equation groups $E_{\textrm{A}}$ and $E_{\textrm{B}}$. $V_{\textrm{ws}, E_{\textrm{A}}}$ and $V_{\textrm{ws}, E_{\textrm{B}}}$ are the within-shared variables of $E_{\textrm{A}}$ and $E_{\textrm{B}}$, and $V_{\textrm{bs}}$ is the between-shared variables of the two equation groups.

Figure 2 shows an example of two equivalent equation groups and their within-group shared variables and between-group shared variables. Our proposed algorithm eliminates the variable *k* to transform the sets of the variables in these equation groups into the same, checks whether each equation in $E_{\textrm{A}}$ is equivalent to an equation in $E_{\textrm{B}}$, and judges they are equivalent.

### Experimental settings

We created 50 equivalent and non-equivalent pairs of equations and equation groups based on physical models in the textbook about process control^22^ to evaluate the proposed method. Tables 1 and 2 present 7 cases, and the entire cases can be found as Supplementary Information. The equations used in the experiments are DAEs consisting of four arithmetic operations, elementary functions, and derivatives.

**Table 1:** Pairs of equations used in experiments.

Case	Equation		Equivalent
1	$E_{\textrm{A}}$	$$\begin{aligned} \rho \dfrac{\textrm{d}V}{\textrm{d}t} = w_1 + w_2 - w \end{aligned}$$	No
	$E_{\textrm{B}}$	$$\begin{aligned}\rho \dfrac{\textrm{d}Vx}{\textrm{d}t} = w_1x_1 + w_2x_2 - wx \end{aligned}$$	
2	$E_{\textrm{A}}$	$$\begin{aligned} \rho \dfrac{\textrm{d}V}{\textrm{d}t} = w_1 + w_2 - w \end{aligned}$$	Yes
	$E_{\textrm{B}}$	$$\begin{aligned} w_1 + w_2 - w - \rho \dfrac{\textrm{d}V}{\textrm{d}t} = 0 \end{aligned}$$	
3	$E_{\textrm{A}}$	$$\begin{aligned} \rho \dfrac{\textrm{d}V}{\textrm{d}t} = w_1 + w_2 - w \end{aligned}$$	No
	$E_{\textrm{B}}$	$$\begin{aligned} \rho \dfrac{\textrm{d}V}{\textrm{d}t} = w_1 - w_2 - w \end{aligned}$$

**Table 2:** Pairs of equation groups used in experiments.

Case	Equation group		Equivalent
4	$E_{\textrm{A}}$	$$\begin{aligned} - r_{\textrm{A}}&= k_0 \exp \left( -E/(RT) \right) C_{\textrm{A}} \\ V\frac{\textrm{d}C_{\textrm{A}}}{\textrm{d}t}&= q (C_0 - C_{\textrm{A}}) + r_{\textrm{A}} V \\ V \rho C \frac{\textrm{d}T}{\textrm{d}t}&= w C (T_{\textrm{i}} - T) + H_{\textrm{r}} V r_{\textrm{A}} + U A (T_{\textrm{c}} - T) \end{aligned}$$	Yes
	$E_{\textrm{B}}$	$$\begin{aligned} -r_{\textrm{A}}&= kC_{\textrm{A}} \\ k&= k_0 \exp \left( -E/(RT) \right) \\ V\frac{\textrm{d}C_{\textrm{A}}}{\textrm{d}t}&= q (C_0 - C_{\textrm{A}}) + r_{\textrm{A}} V \\ V \rho C \frac{\textrm{d}T}{\textrm{d}t}&= w C (T_{\textrm{i}} - T) + H_{\textrm{r}} V r_{\textrm{A}} + U A (T_{\textrm{c}} - T) \end{aligned}$$	
5	$E_{\textrm{A}}$	$$\begin{aligned} A \frac{\textrm{d}h}{\textrm{d}t}&= q_{\textrm{i}} - C_{\textrm{v}} \sqrt{h} \\ C_{\textrm{v}}&= C_0 \sqrt{g/g_{\textrm{c}}} \end{aligned}$$	Yes
	$E_{\textrm{B}}$	$$\begin{aligned} P&= P_{\textrm{a}} + \rho g h /g_{\textrm{c}} \\ q&= C_0 \sqrt{(P - P_{\textrm{a}})/{\rho }} \\ A \frac{\textrm{d}h}{\textrm{d}t}&= q_{\textrm{i}} - q \end{aligned}$$	
6	$E_{\textrm{A}}$	$$\begin{aligned} - r_{\textrm{A}}&= k_0 \exp \left( -E/(RT) \right) C_{\textrm{A}} \\ V\frac{\textrm{d}C_{\textrm{A}}}{\textrm{d}t}&= q (C_0 - C_{\textrm{A}}) + r_{\textrm{A}} V \\ V \rho C \frac{\textrm{d}T}{\textrm{d}t}&= w C (T_{\textrm{i}} - T) + H_{\textrm{r}} V r_{\textrm{A}} + U A (T_{\textrm{c}} - T) \end{aligned}$$	No
	$E_{\textrm{B}}$	$$\begin{aligned} A \frac{\textrm{d}h}{\textrm{d}t}&= q_{\textrm{i}} - C_{\textrm{v}} \sqrt{h} \\ C_{\textrm{v}}&= C_0 \sqrt{g/g_{\textrm{c}}} \end{aligned}$$	
7	$E_{\textrm{A}}$	$$\begin{aligned} -r_{\textrm{A}}&= kC_{\textrm{A}} \\ k&= k_0 \exp \left( -E/(RT) \right) \\ V\frac{\textrm{d}C_{\textrm{A}}}{\textrm{d}t}&= q (C_0 - C_{\textrm{A}}) + r_{\textrm{A}} V \\ V \rho C \frac{\textrm{d}T}{\textrm{d}t}&= w C (T_{\textrm{i}} - T) + H_{\textrm{r}} V r_{\textrm{A}} + U A (T_{\textrm{c}} - T) \end{aligned}$$	No
	$E_{\textrm{B}}$	$$\begin{aligned} -r_{\textrm{A}}&= kC_{\textrm{A}}^2 \\ k&= k_0 \exp \left( -E/(RT)\right) \\ V\frac{\textrm{d}C_{\textrm{A}}}{\textrm{d}t}&= q (C_0 - C_{\textrm{A}}) + r_{\textrm{A}} V \\ V \rho C \frac{\textrm{d}T}{\textrm{d}t}&= w C (T_{\textrm{i}} - T) + H_{\textrm{r}} V r_{\textrm{A}} + U A (T_{\textrm{c}} - T) \end{aligned}$$	

We implemented our proposed algorithm in Python using a Python-based CAS, Sympy^23^. We prepared TeX-formatted equation groups and parsed them using the ‘parse_latex’ function. Although the ‘parse_latex’ function is incomplete, we confirmed that all equations were converted correctly to Sympy expressions.

## Results and discussion

The proposed method correctly judged the equivalence of all 50 pairs. This section describes how the proposed algorithm realized the correct judgment in each case.

### Cases of equation equivalence judgment

In Case 1, the sets of variables of the two equations were different; thus, Algorithm 1 returned false (Lines 1 and 2).

The two equations in Case 2 had the same set of variables and the same solutions for one variable, for example, $w_1$; thereby, Algorithm 1 returned true (Lines 9 and 10).

In Case 3 where the two equations had the same set of variables but the solutions for one variable were different, Algorithm 1 returned false (Lines 11 and 12).

Algorithm 1 fails to accurately judge the equivalence of equations in the following two cases: 1) when the number of the solutions for one variable is more than one and 2) when either equation cannot be solved. The first case occurs when all variables in an equation appear in a second or higher-order form. Since such equations have been rarely seen in describing physical models, they would not be a problem in practice. The second case appears when an equation consists only of partial derivatives of variables, where it is impossible to solve for a single variable without information other than the equation. Developing a method to deal with such cases is a subject for future work.

### Cases of equation group equivalence judgment

In Case 4, the sets of variables of the two equation groups $V_{\textrm{A}}$ and $V_{\textrm{B}}$ were different. Algorithm 3 first derived the between-shared variables and within-shared variables of the two equation groups as follows (Lines 1–3):

1 $$\begin{aligned} V_{\textrm{bs}}&= \{r_{\textrm{A}}, k_{0}, E, R, T, C_{\textrm{A}}, V, t, q, C_0, \rho , C, w, T_{\textrm{i}}, H_{\textrm{r}}, U, T_{\textrm{c}}\}, \end{aligned}$$

2 $$\begin{aligned} V_{\textrm{ws}, E_{\textrm{A}}}&= \{ r_{\textrm{A}}, T, C_{\textrm{A}}, V, t \}, \end{aligned}$$

3 $$\begin{aligned} V_{\textrm{ws}, E_{\textrm{B}}}&= \{ r_{\textrm{A}}, k, T, C_{\textrm{A}}, V, t \}. \end{aligned}$$

Then, the variable appearing only in $E_{\textrm{B}}$, $V_{E_{\textrm{B}}}^* = \{ k \}$ was obtained (Line 5), and *k* in $E_{\textrm{B}}$ was eliminated by substituting $k= k_0 \exp \left( -E/(RT) \right)$ into $-r_{\textrm{A}}=k C_{\textrm{A}}$ (Lines 13–15). After this variable elimination, $V_{\textrm{A}}$ and $V_{\textrm{B}}$ became the same, and all equations in $E_{\textrm{A}}$ were equivalent to those in $E_{\textrm{B}}$; thus, Algorithm 3 returned true (Lines 20 and 21).

In Case 5, $E_{\textrm{A}}$ and $E_{\textrm{B}}$ had different sets of variables, and variable elimination was required in both equation groups. As the same method in Case 4, Algorithm 3 eliminated the variables in two equation groups (Lines 9–18) and returned true (Lines 20 and 21).

Case 6 had two equation groups with different variables. However, the variables appearing only in either equation group were not equal to the variable to be eliminated: for example, $k_0$ appearing only in $E_{\textrm{A}}$ could not be eliminated. Hence, $E_{\textrm{A}}$ and $E_{\textrm{B}}$ were judged non-equivalent (Lines 6 and 7).

In Case 7, the equation groups had different variables, and the sets of variables became the same after variable elimination. The first equation in $E_{\textrm{A}}$ was not equivalent to any equation in $E_{\textrm{B}}$; thereby Algorithm 3 returned false (Lines 22 and 23).

Algorithm 3 sometimes fails to precisely judge the equivalence when an equation group does not lead to a single form after variable elimination. Assuming that we have an equation group as follows and $k_1$ needs to be eliminated.

4 $$\begin{aligned} \begin{aligned} k_1&= k_{10} \exp \left( - E_{1} / (RT)\right) ,\\ k_2&= k_{20} \exp \left( - E_{2} / (RT)\right) ,\\ \frac{\textrm{d}x_{1}}{\textrm{d}t}&= -k_{1} x_{1}, \\ \frac{\textrm{d}x_{2}}{\textrm{d}t}&= -k_{1} x_{1} - k_{2} x_{2}. \end{aligned} \end{aligned}$$

The number of the solutions for $k_1$ is three, and the equation groups after eliminating $k_1$ depend on the solution used for the substitution. Although such examples will need to be addressed in the future, the proposed method is useful for judging the equivalence of many types of physical models as shown in Supplementary Information. (Table S1)

## Conclusion

We proposed a simple rule-based method for equation group equivalence judgment. The proposed method eliminates variables that appear only in either equation group and checks whether all equations in the two equation groups have a one-to-one relationship. The method was implemented in Python and a Python-based CAS, Sympy, and 50 equivalent and non-equivalent pairs of equations and equation groups were used for experiments. The results have shown that the proposed method can accurately judge whether two equation groups are equivalent.

The proposed method still has some limitations. The method has two assumptions to be removed: (1) any equation is solvable for one variable, and (2) the number of solutions for each equation is one. Furthermore, the equivalence judgment algorithm for equation groups (Algorithm 3) cannot correctly judge the equivalence of some equation groups that do not lead to a single form after variable elimination as explained in Section 3.2. Our future work will address these limitations by extending the method in this paper. Furthermore, we plan to expand the scope to more types of calculations, such as summation symbol $\Sigma$, integral symbol $\int$, vectors, and matrices.

## Supplementary Information

Supplementary Information.

## Data Availability

The datasets used in the current study are available at https://github.com/humansys-lab/dae-equiv-judge.

## Code availability

The code used in the current study is available at https://github.com/humansys-lab/dae-equiv-judge.
