# Supplementary material for: Simple algorithm for judging equivalence of differential-algebraic equation systems
Source: Sci Rep. 2023 Jul 17;13:11534. doi: 10.1038/s41598-023-38254-y (PMC10352375; doi:10.1038/s41598-023-38254-y)
Supplement: Supplementary file 1 — Supplementary Information. [file 41598_2023_38254_MOESM1_ESM.pdf]

Simple Algorithm for Judging Equivalence of Differential-Algebraic Equation Systems

Shota Kato, Chunpu Zhang, and Manabu Kano

Department of Systems Science, Kyoto University,
Yoshida-honmachi, Sakyo-ku, Kyoto, 606-8501, Japan

Supplementary Information

$$a = b \tag{S1}$$

$$a - b = 0 \tag{S2}$$

$$a + b = 0 \tag{S3}$$

$$\exp(\frac{b}{a}) = c \tag{S4}$$

$$\frac{b}{a} = \ln c \tag{S5}$$

$$\exp(\frac{a}{b}) = c \tag{S6}$$

$$\begin{aligned} a &= b \\ c &= d \end{aligned} \tag{S7}$$

$$\begin{aligned} a - b &= 0 \\ c - d &= 0 \end{aligned} \tag{S8}$$

$$\begin{aligned} a - b &= 0 \\ c + d &= 0 \end{aligned} \tag{S9}$$

$$\rho \frac{dV(t)}{dt} = w_1 + w_2 - w \tag{S10}$$

$$w_1 + w_2 - w - \rho \frac{dV(t)}{dt} = 0 \tag{S11}$$

$$\rho \frac{dV(t)x(t)}{dt} = w_1 x_1 + w_2 x_2 - wx(t) \tag{S12}$$

$$\rho \frac{dV(t)}{dt} = w_1 - w_2 - w \tag{S13}$$

$$\begin{aligned} \rho \frac{dV(t)}{dt} &= w_1 + w_2 - w \\ \rho V(t) \frac{dx(t)}{dt} &= w_1[x_1 - x(t)] + w_2[x_2 - x(t)] \end{aligned} \tag{S14}$$

$$\begin{aligned} \frac{dV(t)}{dt} &= \frac{1}{\rho}[w_1 + w_2 - w] \\ \frac{dx(t)}{dt} &= \frac{w_1}{\rho V(t)}[x_1 - x(t)] + \frac{w_2}{\rho V(t)}[x_2 - x(t)] \end{aligned} \tag{S15}$$

$$\begin{aligned} A \frac{dh(t)}{dt} &= q_i - C_v \sqrt{h(t)} \\ C_v &= C_0 \sqrt{g/g_c} \end{aligned} \tag{S16}$$

$$\begin{aligned} P &= P_a + \frac{\rho g}{g_c} h(t) \\ q &= C_0 \sqrt{\frac{P - P_a}{\rho}} \\ A \frac{dh(t)}{dt} &= q_i - q \end{aligned} \tag{S17}$$

$$\begin{aligned} k &= k_0 \exp(-\frac{E}{RT(t)}) \\ V \frac{d}{dt} C_A(t) &= q[C_0 - C_A(t)] - VkC_A(t) \\ V\rho C \frac{d}{dt} T(t) &= wC[T_i - T(t)] + (-H_r)VkC_A(t) + UA[T_c - T(t)] \end{aligned} \tag{S18}$$

$$\begin{aligned} -r_A &= k_0 \exp(-\frac{E}{RT(t)}) C_A(t) \\ V \frac{d}{dt} C_A(t) &= q[C_0 - C_A(t)] + r_A V \\ V\rho C \frac{d}{dt} T(t) &= wC[T_i - T(t)] + H_r V r_A + UA[T_c - T(t)] \end{aligned} \tag{S19}$$

$$\begin{aligned} -r_A &= kC_A(t) \\ k &= k_0 \exp(-\frac{E}{RT(t)}) \\ V \frac{d}{dt} C_A(t) &= q[C_0 - C_A(t)] + r_A V \\ V\rho C \frac{d}{dt} T(t) &= wC[T_i - T(t)] + H_r V r_A + UA[T_c - T(t)] \end{aligned} \tag{S20}$$

$$
\begin{aligned}
-r_A &= kC_A(t) \\
k &= k_0 \exp(\frac{-E}{RT(t)}) \\
Q &= \frac{aF_c^{b+1}}{F_c + (\frac{aF_c^b}{2\rho_c c_p})}(T(t) - T_c) \\
\frac{d}{dt}C_A(t) &= \frac{F}{V}[C_0 - C_A(t)] + r_A \\
\frac{dT(t)}{dt} &= \frac{F}{V}[T_0 - T(t)] + \frac{h_r}{\rho C_p} r_A - \frac{Q}{\rho c_p V}
\end{aligned} \tag{S21}
$$

$$
\begin{aligned}
k &= k_0 \exp(\frac{-E}{RT(t)}) \\
Q &= \frac{aF_c^{b+1}}{F_c + (\frac{aF_c^b}{2\rho_c c_p})}(T(t) - T_c) \\
\frac{d}{dt}C_A(t) &= \frac{F}{V}[C_0 - C_A(t)] - kC_A(t) \\
\frac{dT(t)}{dt} &= \frac{F}{V}[T_0 - T(t)] - \frac{h_r}{\rho C_p} kC_A(t) - \frac{Q}{\rho c_p V}
\end{aligned} \tag{S22}
$$

$$
\begin{aligned}
Q &= \frac{aF_c^{b+1}}{F_c + (\frac{aF_c^b}{2\rho_c c_p})}(T(t) - T_c) \\
\frac{d}{dt}C_A(t) &= \frac{F}{V}[C_0 - C_A(t)] - k_0 \exp(\frac{-E}{RT(t)})C_A(t) \\
\frac{dT(t)}{dt} &= \frac{F}{V}[T_0 - T(t)] - \frac{h_r}{\rho C_p} k_0 \exp(\frac{-E}{RT(t)})C_A(t) - \frac{Q}{\rho c_p V}
\end{aligned} \tag{S23}
$$

$$
\begin{aligned}
k &= k_0 \exp(-\frac{E}{RT(t)}) \\
V\frac{d}{dt}C_A(t) &= q[C_0 - C_A(t)] - VkC_A(t)^2 \\
V\rho C\frac{d}{dt}T(t) &= wC[T_i - T(t)] + (-H_r)VkC_A(t)^2 + UA[T_c - T(t)]
\end{aligned} \tag{S24}
$$

$$
\begin{aligned}
-r_A &= kC_A(t)^2 \\
k &= k_0 \exp(-\frac{E}{RT(t)}) \\
V\frac{d}{dt}C_A(t) &= q[C_0 - C_A(t)] + r_A V \\
V\rho C\frac{d}{dt}T(t) &= wC[T_i - T(t)] + H_r V r_A + UA[T_c - T(t)]
\end{aligned} \tag{S25}
$$

$$
\begin{aligned}
V\frac{d}{dt}C_A(t) &= q[C_0 - C_A(t)] - Vk_0 \exp(-\frac{E}{RT(t)})C_A(t)^2 \\
V\rho C\frac{d}{dt}T(t) &= wC[T_i - T(t)] + (-H_r)Vk_0 \exp(-\frac{E}{RT(t)})C_A(t)^2 + UA[T_c - T(t)]
\end{aligned} \tag{S26}
$$

$$
\begin{aligned}
\tau &= H/L \\
\delta &= aG/L \\
K &= G/L \\
\tau\frac{d}{dt}x_1(t) &= K[y_f - b] - [1+\delta]x_1(t) + x_2(t) \\
\tau\frac{d}{dt}x_2(t) &= \delta x_1(t) - [1+\delta]x_2(t) + x_3(t) \\
\tau\frac{d}{dt}x_3(t) &= \delta x_2(t) - [1+\delta]x_3(t) + x_f
\end{aligned} \tag{S27}
$$

$$
\begin{aligned}
H\frac{d}{dt}x_1(t) &= G[y_f - b] - [L+aG]x_1(t) + Lx_2(t) \\
H\frac{d}{dt}x_2(t) &= aGx_1(t) - [L+aG]x_2(t) + Lx_3(t) \\
H\frac{d}{dt}x_3(t) &= aGx_2(t) - [L+aG]x_3(t) + Lx_f
\end{aligned} \tag{S28}
$$

$$
\begin{aligned}
V_1\frac{d}{dt}C_1(t) &= q[C_i - C_1(t)] - V_1k_1 \\
V_2\frac{d}{dt}C_2(t) &= q[C_1(t) - C_2(t)] - V_2k_2
\end{aligned} \tag{S29}
$$

$$
\begin{aligned}
-r_1 &= k_1 \\
-r_2 &= k_2 \\
V_1\frac{d}{dt}C_1(t) &= q[C_i - C_1(t)] + r_1V_1 \\
V_2\frac{d}{dt}C_2(t) &= q[C_1(t) - C_2(t)] + r_2V_2
\end{aligned} \tag{S30}
$$

$$
\begin{aligned}
V_1\frac{d}{dt}C_1(t) &= q[C_i - C_1(t)] - V_1k_1C_1(t) \\
V_2\frac{d}{dt}C_2(t) &= q[C_1(t) - C_2(t)] - V_2k_2
\end{aligned} \tag{S31}
$$

$$
\begin{aligned}
-r_1 &= k_1C_1(t) \\
-r_2 &= k_2 \\
V_1\frac{d}{dt}C_1(t) &= q[C_i - C_1(t)] + r_1V_1 \\
V_2\frac{d}{dt}C_2(t) &= q[C_1(t) - C_2(t)] + r_2V_2
\end{aligned} \tag{S32}
$$

$$
\begin{aligned}
V_1\frac{d}{dt}C_1(t) &= q[C_i - C_1(t)] - V_1k_1C_1(t) \\
V_2\frac{d}{dt}C_2(t) &= q[C_1(t) - C_2(t)] - V_2k_2C_2(t) \\
V_3\frac{d}{dt}C_3(t) &= q[C_2(t) - C_3(t)] - V_3k_3C_3(t)
\end{aligned} \tag{S33}
$$

$$
\begin{aligned}
r_1 &= -k_1 C_1(t) \\
r_2 &= -k_2 C_2(t) \\
r_3 &= -k_3 C_3(t) \\
V_1 \frac{d}{dt} C_1(t) &= q[C_i - C_1(t)] + r_1 V_1 \\
V_2 \frac{d}{dt} C_2(t) &= q[C_1(t) - C_2(t)] + r_2 V_2 \\
V_3 \frac{d}{dt} C_3(t) &= q[C_2(t) - C_3(t)] + r_3 V_3
\end{aligned}
\tag{S34}
$$

$$
\begin{aligned}
V_1 \frac{d}{dt} C_1(t) &= q[C_i - C_1(t)] - V_1 k_1 \\
V_2 \frac{d}{dt} C_2(t) &= q[C_1(t) - C_2(t)] - V_2 k_2 C_2(t) \\
V_3 \frac{d}{dt} C_3(t) &= q[C_2(t) - C_3(t)] - V_3 k_3
\end{aligned}
\tag{S35}
$$

$$
\begin{aligned}
r_1 &= -k_1 \\
r_2 &= -k_2 C_2(t) \\
r_3 &= -k_3 \\
V_1 \frac{d}{dt} C_1(t) &= q[C_i - C_1(t)] + r_1 V_1 \\
V_2 \frac{d}{dt} C_2(t) &= q[C_1(t) - C_2(t)] + r_2 V_2 \\
V_3 \frac{d}{dt} C_3(t) &= q[C_2(t) - C_3(t)] + r_3 V_3
\end{aligned}
\tag{S36}
$$

$$
\begin{aligned}
mC \frac{d}{dt} T(t) &= wC[T_i - T(t)] + h_e A_e [T_e(t) - T(t)] \\
m_e C_e \frac{d}{dt} T_e(t) &= Q - h_e A_e [T_e(t) - T(t)]
\end{aligned}
\tag{S37}
$$

$$
\begin{aligned}
Q_e &= h_e A_e [T_e(t) - T(t)] \\
mC \frac{d}{dt} T(t) &= wC[T_i - T(t)] + Q_e \\
m_e C_e \frac{d}{dt} T_e(t) &= Q - Q_e
\end{aligned}
\tag{S38}
$$

$$
\begin{aligned}
\frac{d}{dt} T(t) &= \frac{w}{m}[T_i - T(t)] + \frac{h_e A_e}{mC}[T_e(t) - T(t)] \\
\frac{d}{dt} T_e(t) &= \frac{Q}{m_e C_e} - \frac{h_e A_e}{m_e C_e}[T_e(t) - T(t)]
\end{aligned}
\tag{S39}
$$

$$
\begin{aligned}
k_1 &= k_{10} \exp(-E_1/RT) \\
k_2 &= k_{20} \exp(-E_2/RT) \\
\frac{d}{dt} x_1(t) &= -k_1 x_1(t) \\
\frac{d}{dt} x_2(t) &= -k_1 x_1(t) - k_2 x_2(t)
\end{aligned}
\tag{S40}
$$

$$
\begin{aligned}
&\frac{d}{dt}x_1(t) = -k_{10}x_1(t)\exp(-E_1/RT) \\
&\frac{d}{dt}x_2(t) = -k_{10}x_1(t)\exp(-E_1/RT) - k_{20}x_2(t)\exp(-E_2/RT)
\end{aligned}
\tag{S41}
$$

$$
\begin{aligned}
r_1 &= k_1 C_T(t)C_A(t) - k_2 C_D(t)C_E(t) \\
r_2 &= k_3 C_D(t)C_A(t) - k_4 C_M(t)C_E(t) \\
r_3 &= k_5 C_M(t)C_A(t) - k_6 C_G(t)C_E(t) \\
k_i &= a_i \exp(-\frac{E_i}{RT_R}) \\
\frac{d}{dt}C_A(t) &= -(r_1 + r_2 + r_3) \\
\frac{d}{dt}C_T(t) &= -r_1 \\
\frac{d}{dt}C_D(t) &= r_1 - r_2 \\
\frac{d}{dt}C_M(t) &= r_2 - r_3 \\
\frac{d}{dt}C_G(t) &= r_3 \\
\frac{d}{dt}C_E(t) &= r_1 + r_2 + r_3
\end{aligned}
\tag{S42}
$$

$$
\begin{aligned}
k_i &= a_i \exp(-\frac{E_i}{RT_R}) \\
\frac{d}{dt}C_A(t) &= -k_1 C_T(t)C_A(t) + k_2 C_D(t)C_E(t) - k_3 C_D(t)C_A(t) \\
&\quad + k_4 C_M(t)C_E(t) - k_5 C_M(t)C_A(t) + k_6 C_G(t)C_E(t) \\
\frac{d}{dt}C_T(t) &= -k_1 C_T(t)C_A(t) + k_2 C_D(t)C_E(t) \\
\frac{d}{dt}C_D(t) &= k_1 C_T(t)C_A(t) - k_2 C_D(t)C_E(t) - k_3 C_D(t)C_A(t) + k_4 C_M(t)C_E(t) \\
\frac{d}{dt}C_M(t) &= k_3 C_D(t)C_A(t) - k_4 C_M(t)C_E(t) - k_5 C_M(t)C_A(t) + k_6 C_G(t)C_E(t) \\
\frac{d}{dt}C_G(t) &= k_5 C_M(t)C_A(t) - k_6 C_G(t)C_E(t) \\
\frac{d}{dt}C_E(t) &= k_1 C_T(t)C_A(t) - k_2 C_D(t)C_E(t) + k_3 C_D(t)C_A(t) \\
&\quad - k_4 C_M(t)C_E(t) + k_5 C_M(t)C_A(t) - k_6 C_G(t)C_E(t)
\end{aligned}
\tag{S43}
$$

$$
\begin{aligned}
k_i &= a_i \exp(-\frac{E_i}{RT_R}) \\
\frac{d}{dt}C_T(t) &= -k_1 C_O C_T(t) C_A(t) + k_2 C_O C_D(t) C_E(t) \\
\frac{d}{dt}C_D(t) &= k_1 C_O C_T(t) C_A(t) - k_2 C_O C_D(t) C_E(t) - k_3 C_O C_D(t) C_A(t) + k_4 C_O C_M(t) C_E(t) \\
\frac{d}{dt}C_M(t) &= k_3 C_O C_D(t) C_A(t) - k_4 C_O C_M(t) C_E(t) - k_5 C_O C_M(t) C_A(t) + k_6 C_O C_G(t) C_E(t) \\
\frac{d}{dt}C_G(t) &= k_5 C_O C_M(t) C_A(t) - k_6 C_O C_G(t) C_E(t) \\
\frac{d}{dt}C_A(t) &= -k_1 C_O C_T(t) C_A(t) + k_2 C_O C_D(t) C_E(t) - k_3 C_O C_D(t) C_A(t) \\
&\quad + k_4 C_O C_M(t) C_E(t) - k_5 C_O C_M(t) C_A(t) + k_6 C_O C_G(t) C_E(t) \\
\frac{d}{dt}C_E(t) &= k_1 C_O C_T(t) C_A(t) - k_2 C_O C_D(t) C_E(t) + k_3 C_O C_D(t) C_A(t) \\
&\quad - k_4 C_O C_M(t) C_E(t) + k_5 C_O C_M(t) C_A(t) - k_6 C_O C_G(t) C_E(t)
\end{aligned} \tag{S44}
$$

$$
\begin{aligned}
r_1 &= k_1 C_O C_T(t) C_A(t) \\
r_2 &= k_2 C_O C_D(t) C_E(t) \\
r_3 &= k_3 C_O C_D(t) C_A(t) \\
r_4 &= k_4 C_O C_M(t) C_E(t) \\
r_5 &= k_5 C_O C_M(t) C_A(t) \\
r_6 &= k_6 C_O C_G(t) C_E(t) \\
k_i &= a_i \exp(-\frac{E_i}{RT_R}) \\
\frac{d}{dt}C_T(t) &= -r_1 + r_2 \\
\frac{d}{dt}C_D(t) &= r_1 - r_2 - r_3 + r_4 \\
\frac{d}{dt}C_M(t) &= r_3 - r_4 - r_5 + r_6 \\
\frac{d}{dt}C_G(t) &= r_5 - r_6 \\
\frac{d}{dt}C_A(t) &= -r_1 + r_2 - r_3 + r_4 - r_5 + r_6 \\
\frac{d}{dt}C_E(t) &= r_1 - r_2 + r_3 - r_4 + r_5 - r_6
\end{aligned} \tag{S45}
$$

Supplementary Table S1: Pairs of equation groups (EGs) used in experiments. Label indicates whether the two EGs are equivalent (1) or not (0). The cases 1–7 in Table 1 and 2 are the same as cases 29, 4, 30, 8, 6, 32, and 36 , respectively.

Case	EG1	EG2	Label	Case	EG1	EG2	Label
1	(S1)	(S2)	1	26	(S1)	(S3)	0
2	(S4)	(S5)	1	27	(S4)	(S6)	0
3	(S7)	(S8)	1	28	(S7)	(S9)	0
4	(S10)	(S11)	1	29	(S10)	(S12)	0
5	(S14)	(S15)	1	30	(S14)	(S10)	0
6	(S16)	(S17)	1	31	(S18)	(S21)	0
7	(S18)	(S19)	1	32	(S18)	(S24)	0
8	(S19)	(S20)	1	33	(S19)	(S22)	0
9	(S21)	(S22)	1	34	(S19)	(S25)	0
10	(S22)	(S23)	1	35	(S20)	(S23)	0
11	(S21)	(S23)	1	36	(S20)	(S26)	0
12	(S24)	(S25)	1	37	(S21)	(S24)	0
13	(S25)	(S26)	1	38	(S22)	(S25)	0
14	(S24)	(S26)	1	39	(S23)	(S26)	0
15	(S27)	(S28)	1	40	(S29)	(S31)	0
16	(S29)	(S30)	1	41	(S29)	(S32)	0
17	(S31)	(S32)	1	42	(S30)	(S31)	0
18	(S33)	(S34)	1	43	(S30)	(S32)	0
19	(S35)	(S36)	1	44	(S33)	(S35)	0
20	(S37)	(S38)	1	45	(S33)	(S36)	0
21	(S38)	(S39)	1	46	(S34)	(S35)	0
22	(S37)	(S39)	1	47	(S34)	(S36)	0
23	(S40)	(S41)	1	48	(S42)	(S44)	0
24	(S42)	(S43)	1	49	(S42)	(S45)	0
25	(S44)	(S45)	1	50	(S43)	(S45)	0
